# Transferability of robotic console skills by early robotic surgeons: a multi-platform crossover trial of simulation training

**DOI:** 10.1007/s11701-022-01475-w

**Published:** 2022-11-02

**Authors:** Kirsten M. Larkins, Helen M. Mohan, Matthew Gray, Daniel M. Costello, Anthony J. Costello, Alexander G. Heriot, Satish K. Warrier

**Affiliations:** 1 Peter MacCallum Cancer Centre, Melbourne, Victoria Australia; 2 International Medical Robotics Academy, North Melbourne, Victoria Australia

**Keywords:** Robotic surgery, Skills training, Surgical innovation, Surgical education

## Abstract

Robotic surgical training is undergoing a period of transition now that new robotic operating platforms are entering clinical practice. As this occurs, training will need to be adapted to include strategies to train across various consoles. These new consoles differ in multiple ways, with some new vendors using flat screen open source 3D enhanced vision with glasses and differences in design will require surgeons to learn new skills. This process has parallels with aviation credentialling across different aircraft described as type rating. This study was designed to test the hypothesis that technical robotic console operating skills are transferrable across different robotic operating platforms. Ten participants sequentially completed four Mimic^®^(Surgical Science) simulation exercises on two different robotic operating platforms (DaVinci^®^, Intuitive Surgical and HUGO™ RAS, Medtronic). Ethical approval and informed consent were obtained for this study. Groups were balanced for key demographics including previous robotic simulator experience. Data for simulation metrics and time to proficiency were collected for each attempt at the simulated exercise and analysed. Qualitative feedback on multi-platform learning was sought via unstructured interviews and a questionnaire. Participants were divided into two groups of 5. Group 1 completed the simulation exercises on console A first then repeated these exercises on console B. Group 2 completed the simulated exercises on console B first then repeated these exercises on console A. Group 1 candidates adapted quicker to the second console and Group 2 candidates reached proficiency faster on the first console. Participants were slower on the second attempt of the final exercise regardless of their allocated group. Quality and efficiency metrics and risk and safety metrics were equivalent across consoles. The data from this investigation suggests that console operating skills are transferrable across different platforms. Overall risk and safety metrics are within acceptable limits regardless of the order of progression of console indicating that training can safely occur across multiple consoles contemporaneously. This data has implications for the design of training and certification as new platforms progress to market and supports a proficiency-based approach.

## Introduction

Training in robotic surgery needs to be adapted to accommodate the demand for high-quality, safe, and efficient educational programs training the surgeon to proficiency across multiple specialities. New curricula must incorporate virtual reality and simulation training to achieve competency across basic skills prior to progressing to in vivo operating [1, 2]. These curricula have previously enabled validation of simulation-based training in robotic surgery, with consensus opinion supporting this approach [3–5]. Despite the availability of multiple virtual reality simulators for robotic training [6], currently only the DaVinci Skills^®^ Simulator™ and the dV-Trainer^®^ have face, content, construct, concurrent and predictive validity [7] and Da Vinci technology currently dominates robotic education and training.

The long-anticipated arrival of alternative robotic operating platforms will necessitate a shift in educational priorities. Multiple operating systems with diverse adaptations of technology for both the robotic arms and the operating console will be available in the short term [8, 9]. Specifications including the mode of 3D visualisation, design of the hand control, incorporation of haptic feedback have significant relevance to the operating surgeon. The HUGO™ RAS has now been approved for clinical practice and utilises different console operating ergonomics to existing technology. The HUGO™ RAS system has a novel pistol grip compared to the pincer grip of DaVinci. 3D vision is incorporated into an open console design using 3D glasses and the foot pedal configuration is altered. Each new platform has unique features that will need to be incorporated into training and certification. Recommendations for multiplatform training are reported in the literature [8, 10] however current published standardised training curricula are incomplete and do not address the question of what multi-platform simulation training will entail.

In robotic surgery, the availability of simulation-based objective metrics for defined outcomes and benchmarking can  improve standards of patient safety by facillitating better education prior to in- human surgery [11]. Simulation-based training is correlated with operative performance and improved operative skills [12, 13], and supports this approach [14]. Utilising simulation, proficiency-based progression training permits objective measurements of performance. This approach using objective measurements for proficiency is a substitute for the traditional surgical training method of see one do one teach one [15]. Objective training metrics improve trainee performance, reduce errors, and increases operative procedural skill knowledge compared to the present Halstead- type approach to training [16]. These are shared goals in multi-platform learning therefore proficiency-based progression training can inform simulation design for multi-platform learning focussing on adequate skill acquisition rather than the somewhat haphazard present approach to training opportunities.

Simulation training and concepts of proficiency have many parallels to the aviation industry [17]. The introduction of multiple different airplanes with different cockpit configurations and operational capabilities necessitated a specific approach to credentialling to operate each aircraft. Educational considerations for each specific robotic platform will need to be incorporated into fundamental training curricula. This study assesses transferability of basic console operating skills in a simulation setting across two different robotic operating platforms. Thisdata will help to inform and enhance skill acquisition of trainees utilising experience gained from the aviation industry.

## Methods

This pilot investigation was conducted at the  InternationalMedical Robotics Academy IMRA between February and March 2022. Ethical approval was obtained (institutional reference number: QA2021043). Ten participants were recruited to participate in this investigation on a voluntary basis with informed consent. This investigation was run in parallel with basic robotic console training at the Australian Medical Robotics Academy (Melbourne, Australia). Participants were sourced from all levels of training from pre-training registrar to fellow to ensure accurate representation of training skill levels. Participants had access to two robotic surgical platforms—the DaVinci Trainer (dV-Trainer^®^ Intuitive Surgical, Sunnyvale CA) and the HUGO™ RAS (Medtronic, Minneapolis MN). The robotic operating platforms will be deidentified for this analysis and presentation of results and referred to as Console A or Console B.

On enrolment participants were surveyed to assess previous robotic training and experience. Participants were then allocated to one of two groups based on their previous robotic console operating experience with an equivalent skill level mix across both groups. A pre-course questionnaire was completed by all participants to assess participants’ demographics, self- assessed confidence, and robotic experience.

**Fig. 1:**
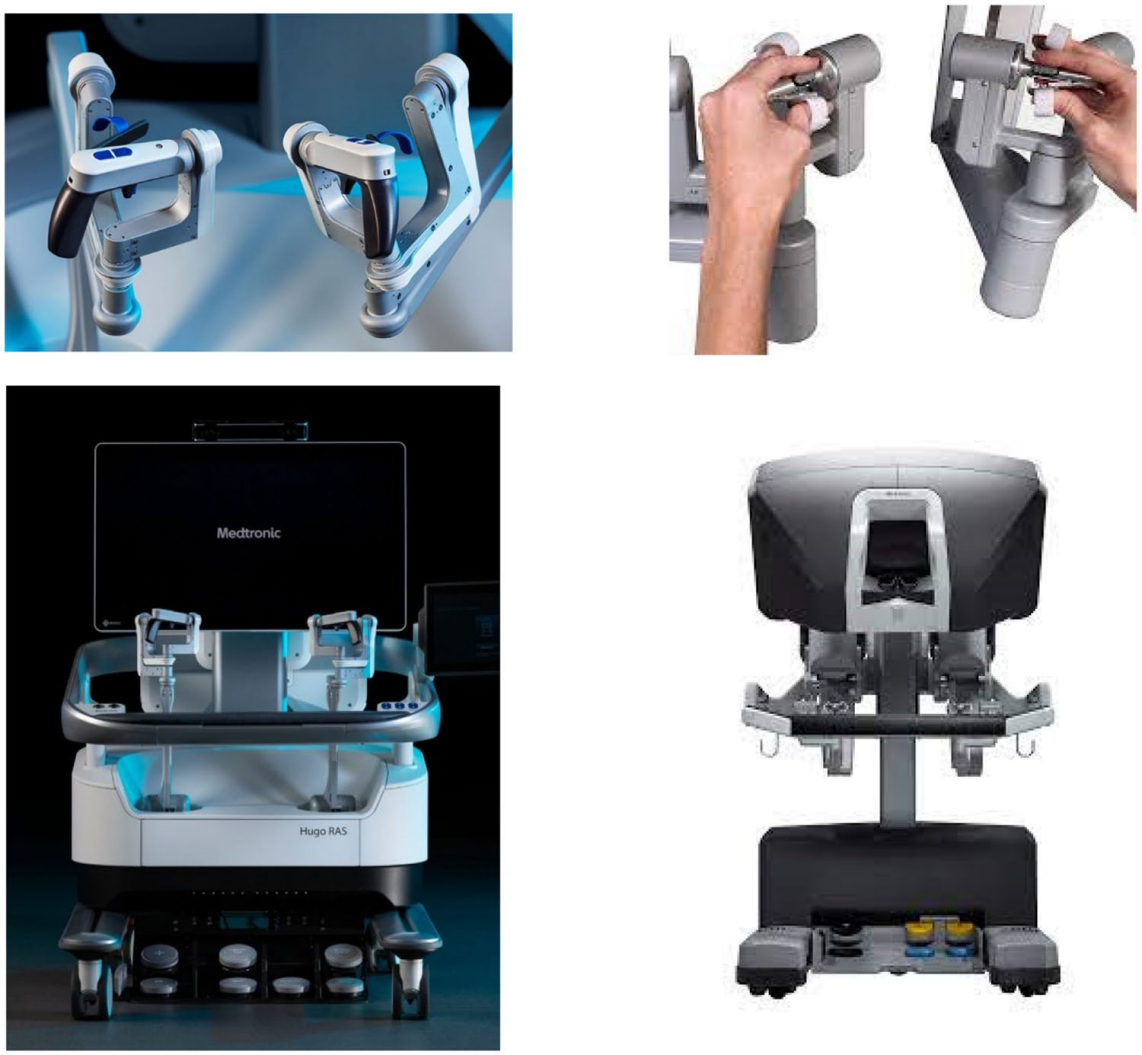
Comparison of robotic consoles

Once allocated, participants completed a set of four selected simulation modules. The simulation activity was performed on both the HUGO™ RAS and the dV-Trainer^®^. On the dV-Trainer® the candidates were exposed to a pincer grip controls and a closed operating console. The HUGO™ RAS candidates were exposed to different console operating conditions with an open console and pistol grip hand controls (Fig. 1)*.*

**Fig. 2:**
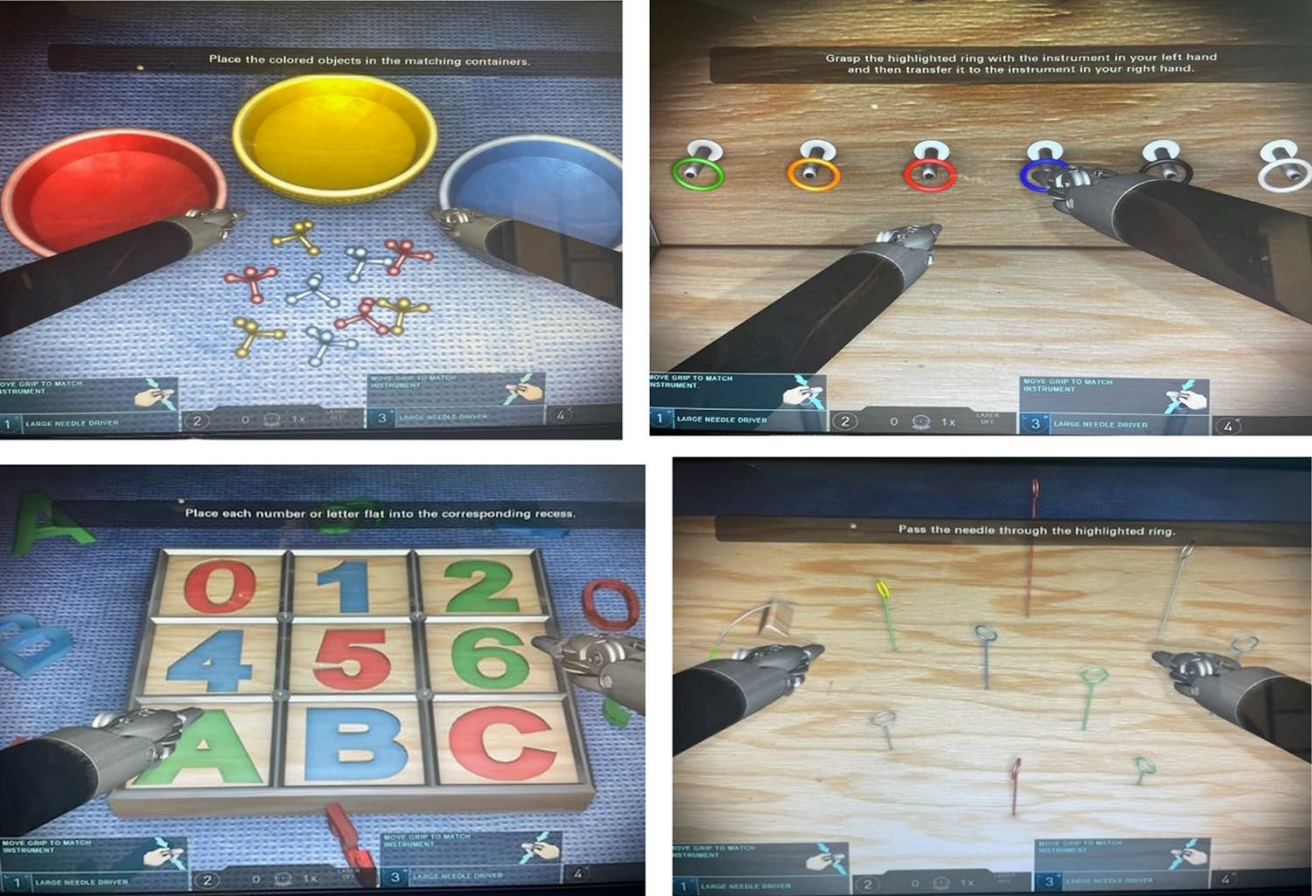
Simulation activities

Buttonology and foot pedal control were also different between the two consoles and candidates were briefly oriented to the console prior to completion of the simulation exercises. Both consoles were linked to the Mimic^®^ Simulation Portal (Mimic Technologies, Surgical Science, Sweden). All simulation exercises were identical in nature and the only variable was the operating console used to complete the exercise*.* The simulated activities selected for comparison were Pick and place, Pegboard 1, Matchboard 1 and Thread the rings 2 (Fig. 2).

**Fig. 3:**
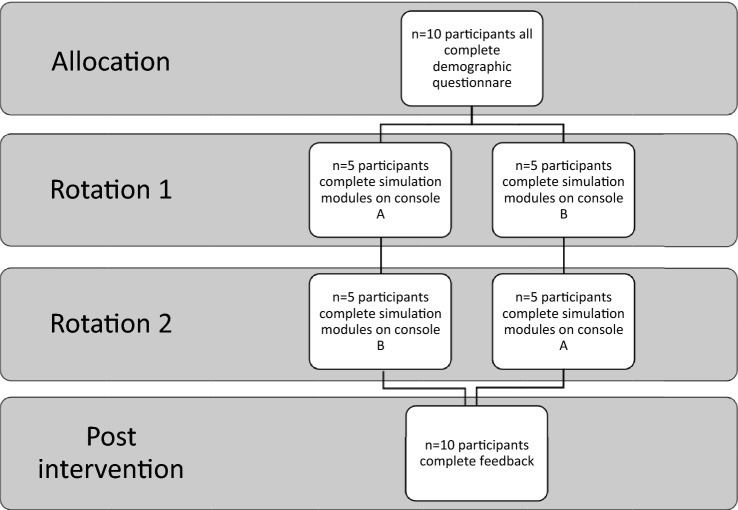
Flow diagram of study protocol

These activities are directly comparable across both consoles with the same metrics set across both consoles for each activity. These activities were selected to provide a graded progression of activities that assessed all essential manipulative and psychomotor abilities—including instrument and camera control, endowrist manipulation, two-handed dexterity and needle driving [18]. To achieve proficiency for an activity the participant had to complete the exercise and pass the pre-defined outcome metrics on two consecutive attempts. Once the allocated time on console A or console B had elapsed—participants completed the activities on the secondary console following a cross over study design (Fig. 3).

Data was collated in a deidentified format and analysed using SPSS (IBM Corp Armonk, NY). Metrics available for comparison were the quality and efficiency metrics of time to complete exercise, economy of motion and master workspace range and safety and risk metrics of instrument collisions, excessive instrument force, instruments out of view and drops. Additional data was collected for overall score, number of attempts to proficiency, and total time to proficiency.

## Results

### Demographics

Ten participants participated in this investigation. Complete data was available for 7 participants on console A and 8 participants on console B. All participants completed all prescribed activities at least once, missing data were due to participants not reaching proficiency in one or more of the simulation activities. Demographic data for Group 1 and Group 2 is summarised in Table 1.

**Table 1:** Participant demographics

Group 1	Group 2
Gender	
Male *n* = 3	Male—*n* = 3
Female *n* = 2	Female**—***n* = 2
Age	
25–30	25–30 *n* = 1
31–35 *n* = 1	31–35 *n* = 2
36–40 *n* = 2	36–40 *n* = 2
40+ *n* = 2	40+
Previous rotation with robotic surgery	
Yes—*n* = 3	Yes**—***n* = 3
No—*n* = 2	No**—***n* = 2
Current access to a robotic platform	
Yes—*n* = 4	Yes**—***n* = 3
No—*n* = 1	No**—***n* = 2
Number of bedside cases	
0–5 *n* = 2	0–5 *n* = 2
6–10	6–10 *n* = 1
11–15 *n* = 1	11–15
16–20	16–20
> 20 *n* = 2	> 20 *n* = 2
Number of simulator hours	
0–5 *n* = 3	0–5 *n* = 3
6–10	6–10 *n* = 1
11–15 *n* = 1	11–15
16–20	16–20
21–25	21–25
26–30 *n* = 1	26–30 *n* = 1
Partial primary operator cases	
Yes—*n* = 0	Yes**—***n* = 2
No—*n* = 5	No**—***n* = 3
Musical instrument	
Yes—*n* = 1	Yes**—***n* = 2
No—*n* = 4	No**—***n* = 3
Video games	
Yes—*n* = 3	Yes**—***n* = 4
No—*n* = 2	No**—***n* = 1

Participants were either general surgical or urology trainees, seven participants were completing fellowship training and three participants were at a pre-vocational training stage of training. Robotic exposure and experience were varied within the group. Six of the ten participants had previously acted as a bedside assistant having completed a rotation where the robot was utilised as part of routine practice. Three participants had not had any access or exposure to robotic surgery in their clinical practice.

Five of the ten participants had completed previous simulation based robotic console training. Simulation activity time ranged from 0 to 30 + hours; however, six out of ten participants had less than 5 h of simulator experience. No participant had completed an in-vivo operation. All previous experience and exposure was with utilising the DaVinci platform.

### Module completion metrics

The mean time to pass each module and mean time to proficiency with mean difference for each module is presented in Table 2. The mean times for Group 1 were on average faster on console B except in the Thread the rings 2 exercise where console A times are faster (Time to pass *A* = 277.38 s vs. *B* = 302.57 and time to proficiency 428.45 s vs 578.14 s). The mean times in Group 2 show tendency towards the first console times being faster except in Matchboard 1 where console A times are faster (Time to pass *B* = 275.21 s vs *A* = 164.14 s and time to proficiency *B* = 477.55 s vs *A* = 348.89 s). This would indicate an easier transition from console A to console B, or less of an initial learning curve on console B rather than a trend towards faster times on the secondary console regardless of which console is used first. There were no statistically significant differences in times between the groups.

**Table 2:** Mean time to complete exercise (pass and proficiency) and mean difference

	*N*	Group 1				*N*	Group 2			
		Mean console A (s)	Mean console B (s)	Mean difference (s)	*p* value		Mean console B (s)	Mean console A (s)	Mean difference (s)	*p* value
Pick and Place										
Time to pass	5	249.01	57.31	191.70	0.192	5	87.95	**130.79**	**− 42.84**	0.372
Time to proficiency	2	394.34	139.45	254.88	0.421	4	118.28	**260.08**	**− 141.80**	0.392
Pegboard 1										
Time to pass	5	158.19	89.87	68.31	0.115	5	91.83	**126.24**	**− 34.41**	0.264
Time to proficiency	3	266.97	170.17	96.80	0.193	3	128.27	**153.74**	**− 25.47**	0.500
Matchboard										
Time to pass	5	182.91	173.52	9.39	0.779	5	275.21	164.14	111.07	0.263
Time to proficiency	5	330.63	**346.89**	**− 16.27**	0.746	4	477.55	348.89	128.65	0.399
Thread the rings 2										
Time to pass	5	227.38	**302.57**	**− 75.19**	0.326	5	201.18	**319.55**	**− 118.37**	0.225
Time to proficiency	3	428.45	**578.14**	**− 149.69**	0.052	4	368.05	**732.80**	**− 364.75**	**0.040**

Bold—time slower on second console

The mean difference of each participant’s time to pass and time to proficiency was analysed to allow for differing levels of prior experience. Thread the rings 2 is the only exercise that shows discriminating power with all other exercises showing no significant difference for time to pass or time to proficiency metrics. There was a statistically significant mean difference in time to proficiency between consoles only for the Thread the rings 2 exercise for the console B first group (*B* = 368.05 s vs *A* = 732.80 s, *p* = 0.040). The mean difference between times to proficiency for the console A first group for this exercise also approaches significance (*A* = 428.45 s vs *B* = 578.14 s, *p* = 0.052). In this exercise there is a discordant association with second console times being slower for both groups (*B* = 368.05 s vs *A* = 732.80 s and *A* = 428.45 s vs *B* = 578.14 s).

### Quality and efficiency metrics

The quality and efficiency metrics of mean economy of motion and mean master workspace range were analysed to assess if there is skill improvement in these metrics with subsequent attempts across consoles. Table 3 shows the mean and mean differences of these metrics. For these metrics the results are not significantly different indicating equivalent performance of both platforms. For the console B group master workspace range was different at a level of statistically significance for pick and place (*B* = 9.37 cm vs *A* = 5.96 cm, *p* = 0.000) indicating adaption to the second platform for this exercise. No other activity reached a statical significance for either group across both economy of motion and master workspace range.

**Table 3:** Quality and efficiency metrics

	Group 1				Group 2			
	Mean console A (cm)	Mean console B (cm)	Mean difference (cm)	*p* value	Mean console B (cm)	Mean console A (cm)	Mean difference (cm)	*p* value
Pick and place								
Economy of motion	153.74	117.60	36.14	0.061	135.46	139.58	− 4.12	0.719
Workspace range	6.12	8.45	− 2.33	0.010	9.37	5.96	3.41	**0.000**
Pegboard 1								
Economy of motion	163.88	161.45	2.42	0.756	186.75	174.61	12.14	0.602
Workspace range	7.18	8.92	− 1.74	0.163	8.86	6.55	2.30	0.103
Matchboard								
Economy of motion	288.14	349.86	− 61.71	0.056	346.12	355.66	− 9.53	0.686
Workspace range	7.18	7.65	− 0.46	0.318	8.08	6.67	1.40	0.275
Thread the rings 2								
Economy of motion	222.06	256.66	− 34.60	0.037	239.83	277.47	− 37.66	0.065
Workspace range	6.24	6.94	− 0.70	0.108	7.38	6.90	0.48	0.378

Bold—significance at 5%

### Risk and safety metrics

Risk and safety metrics of instruments out of view and excessive instrument force were analysed as an average score across attempts as shown in Table 4. The metrics of instrument collisions and drops due to the small sample number were analysed as an absolute risk metric by console—see Table 5.

**Table 4:** Risk and safety metrics: instruments out of view and excessive instrument force

	Group 1				Group 2			
	Mean console A	Mean console B	Mean difference	*p* value	Mean console B	Mean console A	Mean difference	*p* value
Pick and Place								
Instruments out of view	0	0	–	–	0	0	–	–
Excessive force	0	0.2	0.2	0.374	0	0	–	–
Pegboard 1								
Instruments out of view	0.0085	0.0317	**− **0.02317	0.541	0.244	0	0.244	0.374
Excessive force	0	0.012	**− **0.00116	0.374	0	0	–	–
Matchboard								
Instruments out of view	0	0.973	**− **0.973	0.114	3.11	0.0058	3.109	**0.019**
Excessive force	0	0.173	**− **0.0173	0.374	0.0707	0	0.707	0.200
Thread the rings 2								
Instruments out of view	0.451	1.248	**− **0.796	0.225	1.039	2.477	**− **1.437	0.401
Excessive force	0.089	1.238	**− **1.15	0.420	1.522	0.23	1.293	0.127

Bold—significance at 5%

**Table 5:** Risk and safety metrics: total console drops and instrument collisions

	Group 1		Group 2		*p* value
	Mean	Std Dev	Mean	Std Dev	
Drops total console A	1.2	1.64	2.4	2.30	0.371
Drops total console B	2.4	0.19	1.0	1.41	0.264
Collisions total console A	24.40	11.21	33.20	18.46	0.389
Collisions total console B	11.00	4.84	14.80	13.552	0.571

There was no statistically significant difference in the number of drops or instrument collisions when comparing console A and console B. Across the four simulation activities participants were only significantly less likely to have instruments out of view on the matchboard exercise if on console B first (*B* = 3.11 vs *A* = 0.0058, *p* = 0.019). Overall risk and safety metrics were not different across either platform indicating no increased risk of errors with either console.

### Qualitative analysis of survey results

All ten participants felt that multiplatform access was beneficial when completing basic robotic simulation training (very useful *n* = 7, somewhat useful *n* = 3). All participants also felt that the console operating skills were transferrable across platforms to some degree (Absolutely transferrable *n* = 5, somewhat transferrable *n* = 5). Comments on multiplatform training included “very similar platforms with initiation of instruments needs a bit of practice”, “Overall skills very shared across the platforms and overall learning curve decreased with absolute time on any platform as opposed to specific platforms” “Only small adaptations to the basics needed across the platforms, overall, a minor adjustment”. There were no comments or responses that suggested participants felt that they had trouble transferring skills across platforms.

## Discussion

This study introduces the concept of multi-platform robotic training begins the conversation for development of a new curriculum to accelerate skill acquisition in robotic surgery training across different technologies. This study has shown that all candidates when exposed to two different operating consoles in the same training session felt that their skills were transferrable across the operating platforms. The objective metrics of time to pass and time to proficiency did not demonstrate that candidates performed faster with cumulative experience; however, candidate comfort and opinion is a valid consideration. Importantly, there was no change to overall risk when introducing multiple consoles to a learner at the same time. This indicates that multi-platform learning can safely occur.

Multi-platform skills training is an emerging area for study. This is the first published data set directly comparing skill acquisition across different robotic operating platforms. Research limitations field relate to the novel nature of the platforms. As new systems emerge, there is limited data for validation of simulation metrics on new technology and the validity of training approaches which use existing simulation programs. This study has assumed that Mimic® simulation exercises and its metrics from are applicable to the HUGO™ RAS operating system; however, we do acknowledge that this has not been validated. Also of note, while this study represents console skills in a simulated setting, there will be differences in skill requirements with in vivo operating due to the differences in endowrist manipulation and instrumentation, and this has not yet been quantified. This investigation has a small sample size which is a further limitation of the data presented; however, larger studies are currently hampered by the scarcity of and access to novel platforms. The study design of intensive simulation also potentially impacted results through fatigue, with second console times noted to be slower on average by the end of the protocol.

In robotic surgical training, the learning curve has been utilised as a marker of progress and proficiency in procedural skills. Tracking performance improvement across operative case experience has been beneficial in benchmarking performance standards and guiding minimum exposure required to achieve competency [19]. Previous robotic surgical learning curves have identified a trajectory for a specific procedure on a single platform. These platform specific learning curves will be impacted by access to other consoles. This will potentially be seen in changes to the previously observed learning curves due to skill adjustments or skill transference across platforms. Going forward it will need to be determined how access to different consoles impacts on procedure specific learning curves. One method to transition this concept to a multi-platform environment is to use “Combined skills- based” learning curves—which track skill acquisition regardless of which platform is used when operating.

Assessing competency and proficiency is rapidly becoming the future of surgical education [15, 16, 20]. Informed by objective measurements of skill and performance capturing the true essence of skills- based performance is within reach in robotics education through simulation training. The overall goal of Proficiency-based Progression Training is to encourage deliberate practice to achieve mastery. The SIMULATE trial [12] and work through the ORSI robotic training academy [15] currently lead the evaluation of this approach and this study highlights the next steps. With multi-platform learning setting proficiency standards and benchmarking using objective measures of skill and safety by comparing skill acquisition and skill transference are essential. This will include more detailed investigation of the impact of the operational differences between robotic consoles in a clinical context and how access to multiple robotic operating platforms impacts skill acquisition. This should focus on the impact of differing console designs including hand controls.

Within aviation, risk mitigation is a key concept in the training and credentialling of pilots. Like surgical robots, modern airliners are very complicated and complex machines. Type rating training is an approved and standardised course of training which upon successful completion; the pilot is qualified to fly a particular type of aircraft. Training involves a series of simulator exercises and on line training in real time flight. Following this, they enter the recurrent training system where skills, knowledge and techniques are checked and assessed on a regular basis. To ensure maintenance of standards and validation of the training a pilot also undergoes a line check where they are observed flying by a check captain. Integrated within this structure a pilot must maintain “recency” which avoids skills fade and maintains a level of competency [21]. This process is specific for a type of aircraft—although some can be cross licensed if they are similar in operation and function. Aircraft manufacturers such as Boeing and Airbus design aircraft to facilitate as much similarity between their types as possible to keep training costs down and allow flexibility [22]. The decision-making behind what aircraft are deemed similar enough to be cross-licensed is complex and takes into consideration the feel of operation, similarities of the flight deck, performance in test flying for skill transference and similarities in management of critical risks. If significant, differences between aircraft mean pilots cannot be cross licenced to prevent incorrect operation which is particularly important in stressful situations [23].

Robotic operating systems can be likened to aircraft, with similar potential for harm when poorly operated; however, training standards have not yet reached the example set in the aviation industry [17]. Understanding this model of training, however, helps to understand how to train across multiple robotic operating platforms can occur. This study shows risk metrics are unchanged, and performance is similar across the two platforms studied which would indicate that these platforms are not dissimilar enough to cause patient harm if utilised in a combined practice. Individual credentialling for each platform will need to occur; however, based on the data in this study, “type rating” for each robotic type could be set to proficiency rather than a time- based standard. Similarly, concepts of recency (for example number of cases on each platform each year) may need to be explored to ensure patient safety. High-quality randomised data will be the next step in designing a model for multi-platform robotic training programs and practice.

## Conclusion

This investigation is an important first step in informing the design of evidence- based training programs in multi-platform surgical robotic education. The data suggests that overall experience is equivalent regardless of the operating platform the participant is exposed to and shows some degree of robotic console skill transferability. The overall risk and safety metrics are not different across platforms supporting the potential to cross credential based on a proficiency- based model following from the example of aviation simulation. Although a small trial this study highlights the need for further investigation multi-platform learning to assist in improving the efficiency and cost effectiveness of future robotic education.

## Data Availability

The authors listed confirm they have full access to the data to support the publication of this manuscript. Data can be made available for review in a de-identified format if requested.
